# Comparative cardiotoxicity risk of pembrolizumab versus nivolumab in cancer patients undergoing immune checkpoint inhibitor therapy: A meta-analysis

**DOI:** 10.3389/fonc.2023.1080998

**Published:** 2023-03-29

**Authors:** Fabrice Yves Ndjana Lessomo, Zhiquan Wang, Chishimba Mukuka

**Affiliations:** ^1^ Cardiovascular Internal Medicine Department, Zhongnan Hospital of Wuhan University, Wuhan, China; ^2^ Internal Medicine Department, MANSA General Hospital, Mansa, Luapula, Zambia

**Keywords:** cardiotoxicity, meta-analysis, nivolumab, pembrolizumab, risk

## Abstract

**Objective:**

Recently, several researchers have reported the incidence of cardiac-related toxicities occurring with nivolumab (Opdivo) and pembrolizumab (Keytruda). There is still a need for balance between oncology treatment efficacy and reduction of cardiotoxicity burden in immune checkpoint inhibitor (ICI)-treated patients. Thus, the primary aim was to determine whether pembrolizumab or nivolumab would present with a greater risk for cardiotoxicity reports.

**Materials and methods:**

This meta-analysis was performed with respect to the MOOSE reporting guidelines. Studies were retrieved by searching PubMed, Embase, and Google Scholar; the search terms were Keytruda or Pembrolizumab, PD1 inhibitors, anti-PD1 drugs, Nivolumab or Opdivo, and cardiotoxicities or cardiac toxicity. The study was restricted to original articles investigating ICI-induced cardiac immune-related adverse events (irAEs). The targeted population was cancer patients treated with either pembrolizumab or nivolumab monotherapy, of which those with records of any cardiac events following the therapy were labeled as events. The measures used to achieve the comparison were descriptive proportions, probabilities, and meta-analysis pooled odds ratios (ORs).

**Results:**

Fifteen studies were included in this meta-analysis. Nivolumab accounted for 55.7% cardiotoxicity and pembrolizumab, for 27.31% (P = 0.027). The meta-analysis was based on the Mantel–Haenszel method, and the random-effect model yielded a pooled OR = 0.73 (95% CI [0.43–1.23] P = 0.24), with considerable heterogeneity (I^2^ = 99% P = 0). Hence, the difference in cardiotoxicity odds risk between pembrolizumab and nivolumab was not statistically significant. On subgroup analysis based on cardiotoxicity type, the “myocarditis” subgroup in which there was no statistical heterogeneity was associated with a significant cardiotoxicity risk increase with pembrolizumab (OR = 1.30 [1.07;1.59], P< 0.05; I^2^ = 0%, Ph = 0.4).

**Conclusion:**

To our knowledge, this is the first meta-analysis to compare the cardiotoxicity potentials of nivolumab and pembrolizumab. In contrast to previous reports, the overall findings here demonstrated that nivolumab-induced cardiotoxicity was more commonly reported in the literature than pembrolizumab; however, myocarditis seemed more likely to occur with pembrolizumab therapy.

## Graphical Abstract

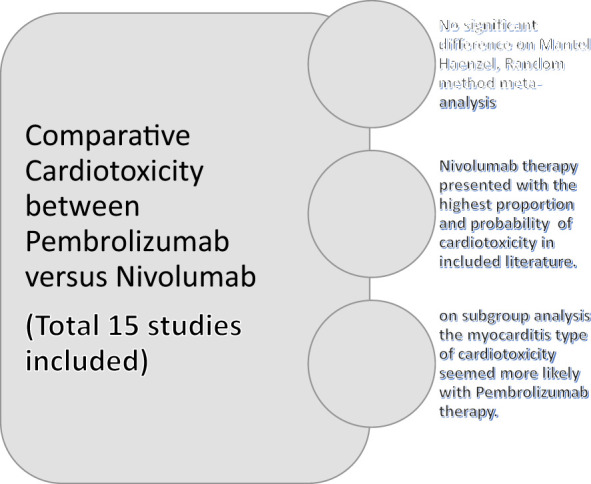

## 1 Background

The World Health Organization’s most recent data from 2021 has shown that cancer in general accounted for around 2 million of the nearly 10 million deaths worldwide (1). Therefore, it might be viewed as a significant health burden that can be reduced through early detection, precise diagnosis, and improved management and care. However, the pharmaceutical agents used to treat cancer, either alone or in combination therapy, have been linked to the emergence of toxicities affecting several vital organs, including the heart and vessels. In the growing field of cardio-oncology, which serves as a link between cardiologists and oncologists, the impact of cancer treatment on the heart and the management of cardiotoxicity are the main topics of interest (1, 2). New therapeutic agents have emerged over the past decade to improve cancer treatment and lessen the toxic side effects, including immune checkpoint inhibitors (ICIs), a special type of immunotherapy subclass whose cardiotoxicity potential was found to be lower than that of conventional chemotherapeutic drugs (3).

The development of these agents has been a revolutionary milestone that was associated with remarkable benefits and resulted in long-lasting tumor responses. They are now widely accepted as a key component of therapeutic strategies in cancer management (4, 5). The ICI subclasses include monoclonal antibodies that block programmed cell death receptors or their ligand (PD-1/PD-L1) and cytotoxic T-lymphocyte-associated antigen 4 (CTLA-4). PD-1, PD-L1, and CTLA-4 immune checkpoints are markedly expressed in cancer cells and contribute to the inhibition of T-cell activation and are thought to represent one of many tumoral adaptive responses to escape from the immune system (6). The first successful use of CTLA-ICI therapy on mice was reported in 1996; then, in 2000, the first human CTLA-4 ICI, i.e., IPILUMAB, was introduced that got FDA approval in 2011 (7, 8). In 2006, nivolumab became the first PD1 ICI used in patients and also the first to obtain FDA approval in 2014 for melanoma (9) followed by pembrolizumab, another PD1 ICI (9). Nevertheless, their use also led to increased occurrences of different types of side effects (irAE), characterized by autoimmune reactions in various tissues that were rare but had serious side effects on the heart (10). In recent years, several studies have consistently reported the incidence of cardiac-related toxicities such as myocarditis, athero-cardiovascular disease, and heart failure in the setting of PD1 ICI therapy among cancer patients (11–14).

The possible theories explaining the rationale of the occurrence of cardiac adverse events with ICI therapy among cancer patients have previously been explored in experimental animal models. It was found that PD1 and PDL1 upregulation had a cardioprotective role for cardiomyocytes; therefore, their blockades or inhibition with PD1 ICI would favor cardiomyocyte damage. Many research papers also reported that there can be an increased likelihood of rare but aggressive cardiotoxic events associated with the use of the PD1 inhibitor subclass of ICI compared with other ICIs. However, because PD1 ICI drugs were found to be safer in terms of the occurrence of other high-grade iRAes, they are being more frequently used than the rest. This gave rise to the need for establishing a balance between this novel oncology treatment efficacy and the burden of drug-adverse cardiac effects among treated patients. However, to our knowledge, no studies have yet been published directly to compare the risk of cardiotoxicity events between nivolumab and pembrolizumab, the two most commonly encountered PD1 ICI drugs used in cancer management. Logically, identification of the drug with a higher tendency for cardiac toxicities than others would be essential to helping specialist caregivers to select the better alternative for patients, taking into account both efficacy and cardiotoxicity indexes in their management and thereby possibly lessening the burden of PD1 ICI-induced cardiotoxicity.

### 1.1 Objectives

The primary aim of this study was to determine by means of a meta-analysis whether pembrolizumab or nivolumab would be associated with increased cardiotoxicity risk.

## 2 Materials and methods

This meta-analysis was conducted in accordance with the MOOSE reporting guidelines (15).

### 2.1 Types of participants

Patients with cancer who were receiving ICI treatment and whose cancer characteristics met the criteria for either pembrolizumab or nivolumab monotherapy, or both, were considered, and those who reported any cardiac pathologies after the start of monotherapy were categorized as events (cases).

### 2.2 Search methods for identification of studies

Relevant literature was obtained by searching PubMed, Embase, and Google Scholar, and an advanced search tool was used to restrict results to only human studies published between 2016 and 1/10/2022. The reference lists of some results were manually retrieved and screened to identify any study that can meet the inclusion criteria.

### 2.3 Electronic searches

All electronic searches were made on the Google Chrome version 105.0.5195.127 software.

The search entry terms were as follows: Keytruda or Pembrolizumab; PD1 inhibitors; anti-PD1 drugs; Nivolumab or Opdivo; cardiotoxicities or cardiac toxicity; and toxicities.

Our initial search strategy on PubMed was (((Keytruda) OR (pembrolizumab)) AND ((cardiotoxicities) OR (cardiac toxicity))) AND (((Opdivo) AND (Nivolumab)) AND ((cardiotoxicities) OR (cardiac toxicity))), which yielded few results. Therefore, another search strategy was implemented using (immune checkpoint inhibitors) AND (cardiotoxicity), which produced more results and was replicated in Embase and Google Scholar as well.

Reports containing the search terms were screened based on the relevance of their title and abstract; eligibility was assessed based on whether the studies addressed the issue of immune checkpoint-related cardiotoxicity. Therefore, irrelevant content was discarded according to the author’s own opinion and purpose. Then, the full-text quality of the remaining articles was assessed to determine whether they would contribute to the study’s aim.

### 2.4 Selection of studies

Study selection was done by two authors and was based on the following criteria:

i) Original studies: including prospective and retrospective studies that reported the cardiotoxicity due to ICI therapy in cancer patients irrespective of the type of cancer.
ii) Significant sample, VigiBase studies including all the cardiotoxicity reports after ICI therapy in cancer patients were also considered.
iii) Eligible studies should have disclosed any cardiac side effects that occurred during the course of pembrolizumab or nivolumab monotherapy.
iv) Eligible studies had to provide basic demographic data on the included participants.

### 2.5 Exclusion criteria

Case reports and case series were excluded because they did not provide the total number of patients treated with pembrolizumab and nivolumab monotherapy.

Because of the comparative nature of this study that targeted PD1 inhibitors, studies providing data only for subclasses of ICI not including PD1 inhibitors or those including only one of the PD1 inhibitors (pembrolizumab or nivolumab) were excluded.

### 2.6 Quality assessment

To assess the quality of each included paper, the Newcastle–Ottawa Scale (NOS) scoring based on 10 points was implemented. A study was considered of good quality when the NOS score was >6; otherwise, it was considered of low quality.

### 2.7 Measures of comparator effect

The outcome of interest was the overall number of reports of cardiac side effects including myocarditis, pericarditis, heart failure, arrhythmia, coronary events, and major adverse cardiovascular events (MACE). For each study, the descriptive proportion of ICI-induced cardiotoxicity attributable to each drug and the probability of developing cardiotoxicity among the subset of patients treated with each drug was determined for description purposes, by the following formula.


$$cardiotoxicity\;proportion\left(x\right)=\frac{number\;of\;events\;imputed\;to\;x}{total\;number\;of\;events\;from\;all\;ICI}$$

$$cardiotoxicity\;probability\left(x\right)=\frac{number\;of\;cardiac\;events\;imputed\;to\;x}{total\;number\;of\;participants\;treated\;with\;x}$$

(Where (x) represents either nivolumab or pembrolizumab)

To compare the means of the cardiotoxicity proportions and probabilities between the two drugs, a one-tailed, P-value Student’s *t*-test for independent samples was used. Given the retrospective nature of the study, the odds ratio on the random and fixed effect modality was estimated with its 95% confidence interval as the outcome measure effect of the meta-analysis (16).

### 2.8 Data extraction and management

Data were collected by the first author and cross-checked by the second author and then extracted into a Microsoft Excel file. For each included study, the collected data for the analyses were as follows: the name of the first author, the year of publication, patients’ mean age (when available), type of malignancy, presence of cardiovascular risk factors, medical history, type of cardiotoxicity reported, total number of cardiotoxicity reports, number of patients treated with pembrolizumab, number of pembrolizumab-induced cardiotoxicity cases, number of patients treated with nivolumab, and its corresponding number of cardiotoxicity-induced cases. If an article did not provide the total number of patients treated for each drug but provided reporting the odds ratio and the number of events (cases), the number of treated patients was estimated by deducting from the ROR and related formulas provided within the study. For vigilance studies, the total number of each drug’s adverse effect was considered as the total number of patients treated and the number of cardiac adverse effects was used as events.

### 2.9 Subgroup analyses

Subgroup analyses were performed to investigate the source of heterogeneity among studies; therefore, studies were grouped according to several criteria:

- According to the grading of the study’s quality: based on their NOS score, studies were grouped into low and good quality.
- According to the report of cardiovascular disease history: grouped as (yes or no).
- According to the type of malignancy: we had the subgroup of studies reporting only on lung cancer (labeled OL) and studies reporting on lung cancer and other cancers (labeled ALL).
- According to the type of cardiotoxicity: we included studies that reported only on myocarditis, and the group reporting and myocarditis plus others such as pericarditis and arrhythmias.

### 2.10 Assessment of risk of publication bias

Publication bias was assessed by a funnel plot associated with Egger’s test.

### 2.11 Sensitivity analysis

The robustness of the findings were explored with the “*cop”* argument in the “*metasens”* package in R statistical software. The Copas selection model described in Copas and Shi (2001) (<DOI: 10.1177/096228020101000402>) evaluates the sensitivity of meta-analysis, helping to determine the possible selection bias.

Statistical analyses were performed with Microsoft Office Excel 2016, and meta‐analysis calculations were achieved with R statistical software version 4.1.3 (2022-03-10) (“One Push-Up” Copyright (C) 2022 The R Foundation for Statistical Computing Platform: i386-w64-mingw32/i386). We used the Cochran’s Q statistic to estimate statistical heterogeneity and the I^2^ statistic to quantify inconsistency. The assumption of homogeneity was considered invalid if P< 0.10.

## 3 Results

### 3.1 Results of the search

**Figure 1:**
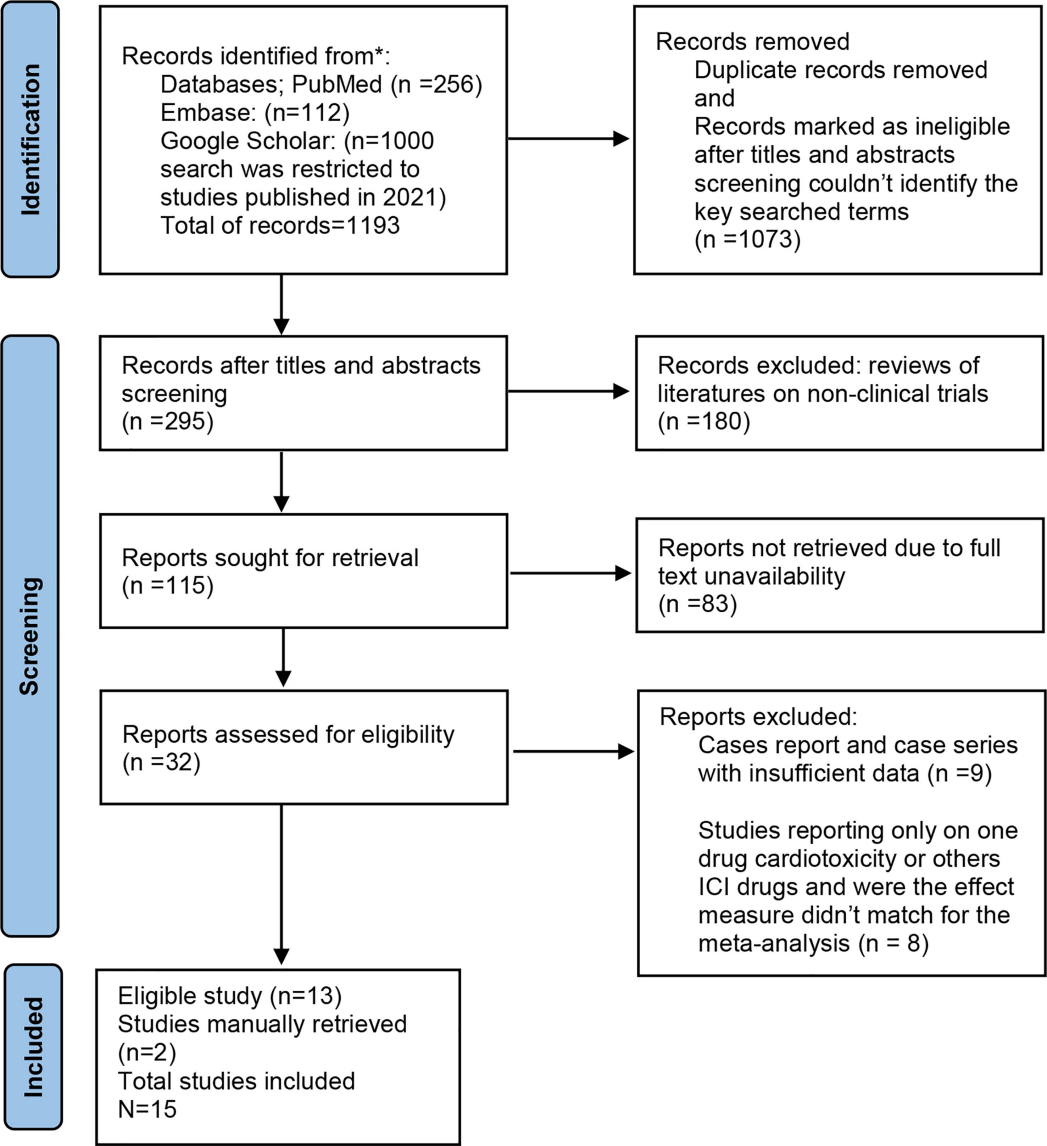
Prisma flow diagram.

The search results are detailed in the flowchart below (**Figure 1**). A total of 1,368 records were identified by searching the database, of which 15 were deemed eligible for this analysis.

For this meta-analysis, a total of 15 observational studies that appeared between 2018 and 2022 were included. The total number of participants in those studies was 7,517,257 (**Table 1**), and the mean age was estimated to be around 66 years. These participants all had malignancies that had been clinically diagnosed and confirmed, such as lung cancers, melanomas, Hodgkin lymphomas, endocrine cancers, and renal cancers, with lung cancers accounting for >60% of all cases (**Table 1**). Anti-PD1, anti-CTLA4, and anti-PDL1 were the immunotherapeutic drug classes reported in each study. Following monotherapy or combination therapy, a total of 18,833 ICI-induced cardiac adverse events were reported.

**Table 1:** Characteristic of included studies.

Study	year	pem_eve	pem_tot	nivo_eve	nivo_tot	NOS_scale	Mean age	CVHX	typ_ca	cardiotoxity	total	C.Reports
**Mahmood &al**	2018	11	52	7	60	6	65	yes	OL	myocarditis	140	35
**Shiori &al**	2020	15	2148	24	4419	8	NA	no	A	others	4419	45
**Serena&al**	2020	7	15	4	8	8	68	yes	A	myocarditis	30	13
**Qian &al**	2019	69	493	125	968	7	65	no	A	myocarditis	43147	315
**Nida&al**	2021	22	123	66	217	8	62	yes	A	others	424	424
**Nestor&al**	2021	1013	25597	2014	46767	5	65	yes	A	others	13646	4401
**Melissa&al**	2020	4	47	19	137	7	64	no	OL	others	196	23
**Joe et al**	2018	43	10321	100	10321	6	NA	no	A	others	3121	1073
**Chitturi&al**	2019	6	45	10	71	10	68	yes	A	others	135	30
**Chenxin &al**	2021	2808	46251	6836	78047	6	NA	no	A	others	7443137	9271
**Anna&al**	2021	805	22378	1076	2791	6	69	no	A	others	2478	2478
**Chenglui &al**	2022	324	2181	196	1766	9	62	yes	A	myocarditis	5518	691
**Zach&al**	2022	17	243	8	220	8	65	yes	A	others	538	26
**Maria&al**	2020	1	8	3	52	8	70	no	OL	others	60	4
**Luke &al**	2021	0	39	2	37	7	71	yes	A	others	268	4
**total**		5145	109941	10490	145881		66.16667				7517257	18833

pem_eve, number of cardiotoxicity report with pembrolizumab as ICI monotherapy; pem_tot, number of patients treated with pembrolizumab; nivo_eve, number of cardiotoxicity reports with nivolumab as ici monotherapy; nivo_tot, patients treated with nivolumab; NOS, Newcasttle Ottawa scale; cvhx, history of cardiovascular disease; typ_ca, type of cancer; OL, only lung cancer, A, lung and others cancers such as melanoma, renal cancer, lymphoma,metastasis; c.reports, total number of ici induced cardiotoxicity cases.

**Figure 2:**
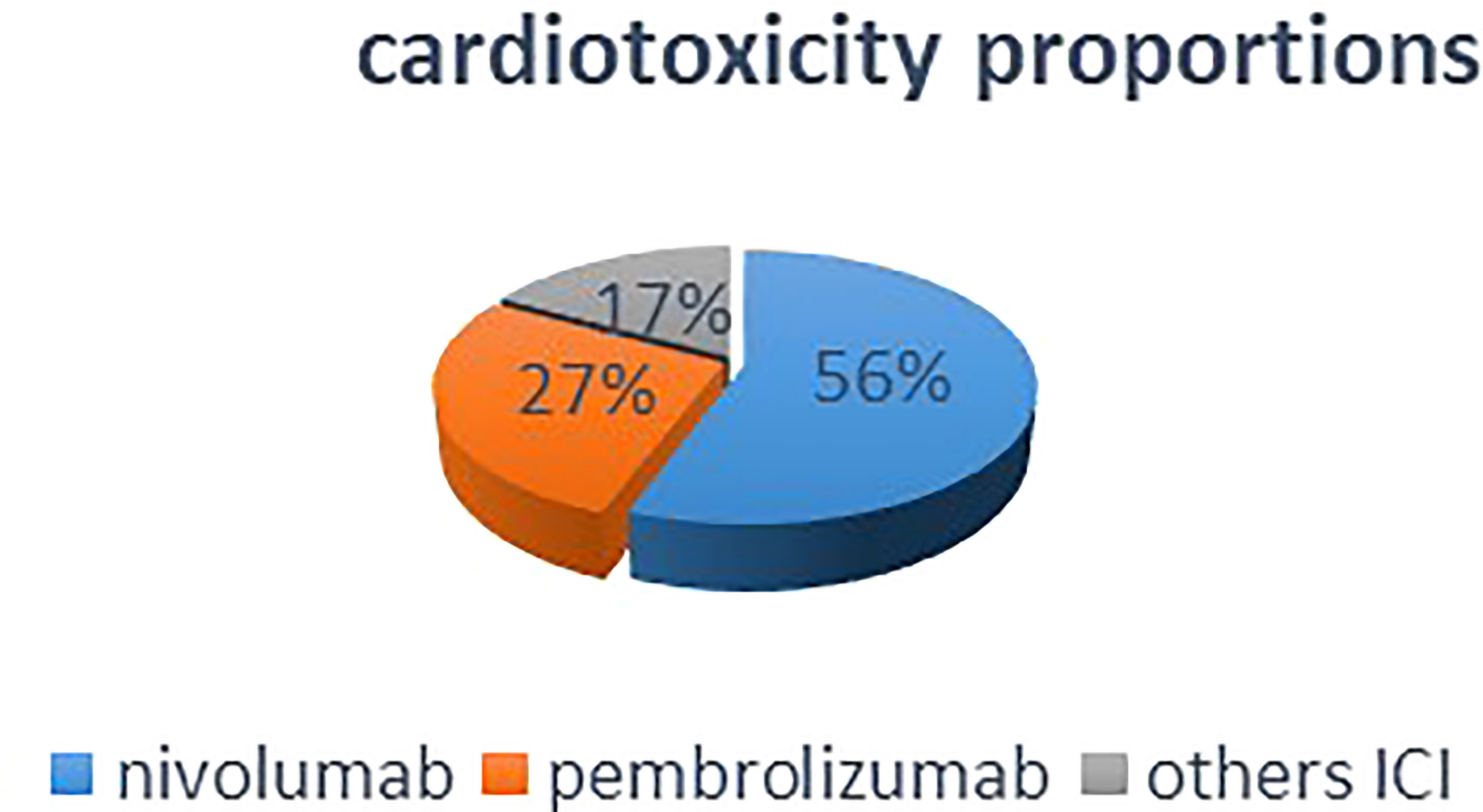
This pie chart shows that in overall included studies, nivolumab monotherapy accounted for half percent of the total ICI-induced cardiotoxicities.

The total number of ICI-induced cardiotoxicity attributable to nivolumab was 55.7%, whereas that for pembrolizumab was for 27.31% (**Figure 2**). The difference in mean cardiotoxicity proportion between nivolumab and pembrolizumab was statistically significant (P = 0.027). Based on the available data, patients treated with pembrolizumab had a 4.6% chance of developing cardiotoxicity after treatment, whereas those treated with nivolumab had a 7.1% chance of developing cardiotoxicity, but the difference in cardiotoxicity probability means between the two drugs was not statistically significant (P = 0.28) (**Table 2**).

**Table 2:** Ici induced cardiotoxicity proportion attributable to pembrolizumab and Nivolumab.

Study	year	pem_eve	pem_tot	nivo_eve	nivo_tot	quality_grade	Study design	total	C.Reports	Propor_P	Propor_N2	PROBP	PROBN
Mahmood &al	2018	11	52	7	60	low	analytic	140	35	0.314286	0.2	0.211538	0.116667
Shiori &al	2020	15	2148	24	4419	good	descriptive	4419	45	0.333333	0.533333	0.006983	0.005431
Serena&al	2020	7	15	4	8	good	analytic	30	13	0.538462	0.307692	0.466667	0.5
Qian &al	2019	69	493	125	968	good	descriptive	43147	315	0.219048	0.396825	0.139959	0.129132
Nida&al	2021	22	123	66	217	good	analytic	424	424	0.051887	0.15566	0.178862	0.304147
Nestor&al	2021	1013	25597	2014	46767	low	descriptive	13646	4401	0.230175	0.457623	0.039575	0.043065
Melissa&al	2020	4	47	19	137	good	analytic	196	23	0.173913	0.826087	0.085106	0.138686
Joe et al	2018	43	10321	100	10321	low	descriptive	3121	1073	0.040075	0.093197	0.004166	0.009689
Chitturi&al	2019	6	45	10	71	good	analytic	135	30	0.2	0.333333	0.133333	0.140845
Chenxin &al	2021	2808	46251	6836	78047	low	descriptive	7443137	9271	0.30288	0.737353	0.060712	0.087588
Anna&al	2021	805	22378	1076	2791	low	descriptive	2478	2478	0.324859	0.434221	0.035973	0.385525
Chenglui &al	2022	324	2181	196	1766	good	analytic	5518	691	0.468886	0.283647	0.148556	0.110985
Zach&al	2022	17	243	8	220	good	analytic	538	26	0.653846	0.307692	0.069959	0.036364
Maria&al	2020	1	8	3	52	good	analytic	60	4	0.25	0.75	0.125	0.057692
Luke &al	2021	0	39	2	37	good	analytic	268	4	0	0.5	0	0.054054
total		5145	109941	10490	145881			7517257	18833	0.273191	0.557001	0.046798	0.071908

Propor_p, cardiotoxicity proportion attributable to pembrolizumab; Propor_N, cardiotoxicity proportion attributable to Nivolumab; PROBP, probability with pembrolizumab; PROBN, probability with Nivolumab.

### 3.2 Meta-analysis results

**Figure 3:**
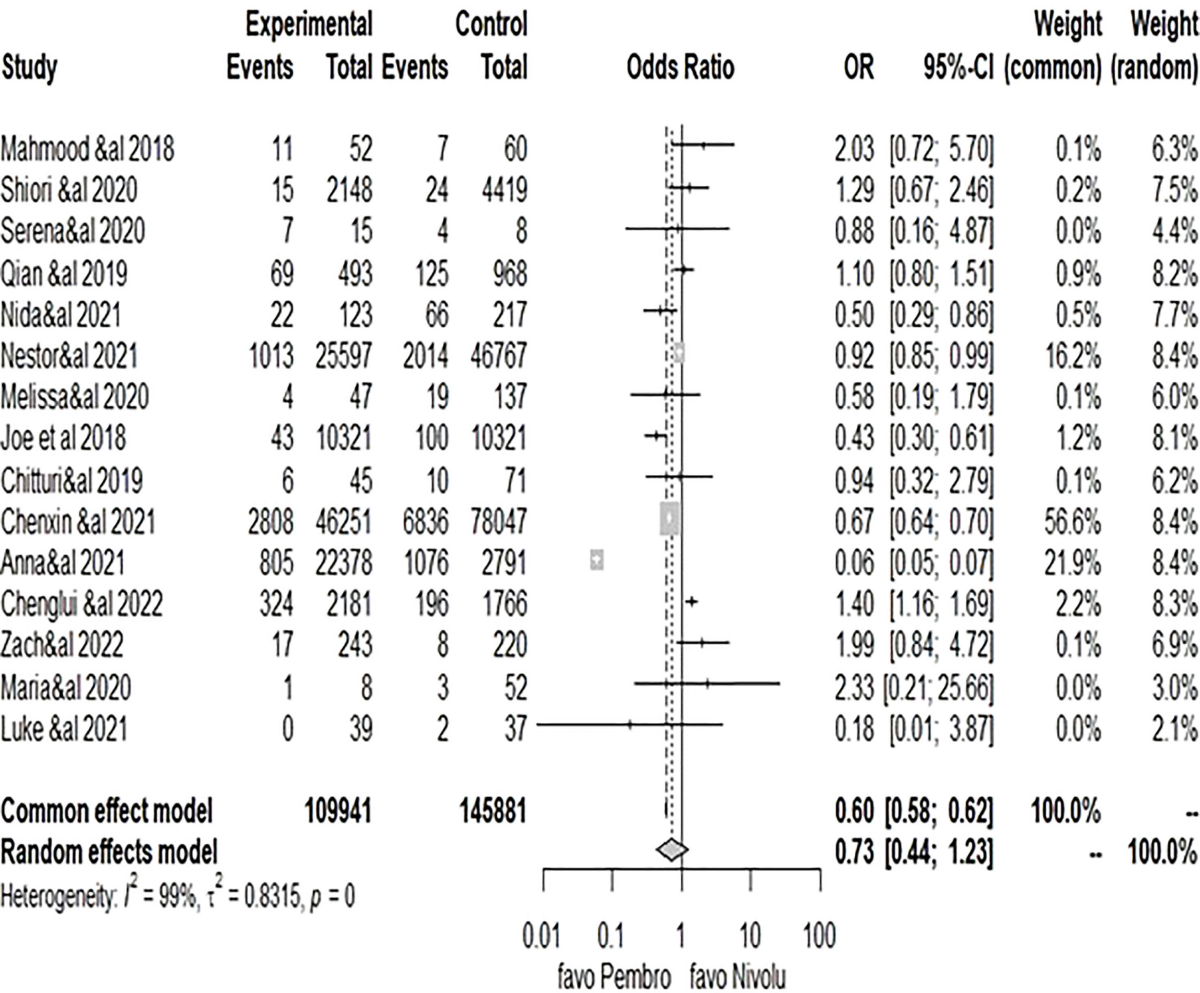
Forest plot of comparison of pembrolizumab vs. nivolumab, outcome: 1.1 cardiotoxicity odds ratio.

The meta-analysis conducted with the recommended Mantel–Haenszel method when explored with the random-effect model for dichotomous outcome variables (16, 17) comparing the cardiotoxicity odds ratio between pembrolizumab and nivolumab yielded a pooled OR of 0.7347 (95% CI: 0.4371–1.2348, P = 0.24) (**Figure 3**), with considerable heterogeneity (I^2^ = 99%, P_h_ = 0.00), showing that the difference between the risk of cardiotoxicity reports between pembrolizumab and nivolumab was not statistically significant. However, it would be significant for pembrolizumab on the common (fixed model) OR = 0.5975 [0.5769–0.6190], P< 0.0001, in favor of pembrolizumab. Unfortunately, the fixed-effect model cannot be considered here because of the wide variability of effect measures among the included studies. Because of the high heterogeneity (I^2^ = 99%), the pooled reporting odds ratio should not be given much consideration. Subgroup analyses were then performed as presented in the next subsection.

### 3.3 Subgroup analyses

Based on the data that was made available, subgroup analyses were carried out according to the following parameters: study quality (good and low); type of malignancy (only lung and all); presence of cardiovascular history or risk factors (yes and no); type of malignancy (myocarditis and other); and subgroup analysis results. High heterogeneity was present in the subgroups of “low quality,” “other cardiotoxicities,” and “all cancers,” whereas moderate-to-low heterogeneity was present in the subgroups of “good quality,” “only lung cancers,” and “myocarditis.” However, apart from the myocarditis group, where a higher risk of cardiotoxicity report was linked to pembrolizumab therapy (OR = 1.30 [1.07–1.59], P< 0.05) (**Table 3** in the annex), the results among other subgroups remained insignificant.

**Table 3:** Summary of meta-analyses and subgroup analysis.

Analysis modality	OR, 95%CI, P-value	I^2^, P_h_	observations
Over all analysis	0.7347 95%CI [0.4371-1.2348] P=0.24 (R.E)	I^2^=99%, P_h_=0	There could be a decrease risk of cardiotoxicity associated with Pembrolizumab than Nivolumab but the association was not statistically significant
Subgroup analysis based on the quality of included studies; Low quality(5 studies included)	OR=0.48[0.15; 1.52], P>0.005 OR=1,04 95%CI[0.75;1.45]	i^2^=100%, P_h_=0 i^2^=50%,Ph=0.04	In the low quality group the cardiotoxicity risk appeared reduced with pembrolizumab but the result was not statistically significant and the heterogeneity was very high with In the good quality subgroup there was a non significant increased of cardiotoxicity with pembrolizumab than Nivloumab and the heterogeneity was moderate and tolerable
Good quality (10 studies included)			
Subgroup analyses based on the type of cancers Only lung Cancer(3 studies included)	OR=1.24[0.46;3.32],P>0.005 OR=0.67[0.37;1.19], P>0.05	i2=31%,Ph=0.24 i2=99%,Ph=0	although the assumption of homogeneity was kept within the (OL) subgroup, the results were still not significant but seemed to favour Nivolumab. Within the (A) subgroup the heterogeneity was considerable and the result remained not significant with tendency to favour Pembrolizumab.
All cancers(12 studies included)			
Subgroup analyses: presence of cardiovascular history YES( 8 studies included)	OR=1,05[0.72;1.63], P>0.05 OR=0.54[0.22;1.31], P>0.05	i2=75%,Ph=0.01 i2=100%,Ph=0.0	Both subgroups did not resolve the heterogeneity issue and still reported a non statistically significant result. The “yes” subgroup favoured Nivolumab while the “No” subgroup favoured Pembrolizumab
NO(7studies included)			
Subgroup analysis: type of cardiotoxicity Myocarditis (4 studies included)	OR=1.30[1.07;1.59], P<0.05 OR=0.59[0.31;1.14], P>0.05	i2=0%,Ph=0.4 i2=100%,Ph=0.0	within the “myocarditis” subgroup the assumption of homogeneity was kept and the result was significant and favoured Nivolumab induced cardiotoxicity
Others			

### 3.4 Results of publication bias assessment

The funnel plot obtained displayed obvious asymmetry that was confirmed by linear regression Egger’s test (P = 0.9963), indicating the strong presence of publication bias among the studies (**Supplementary File**).

### 3.5 Sensitivity analyses

The overall sensitivity analysis revealed that even when selection bias was assumed, the OR and its 95%CI did not vary significantly. The reliability of this meta-analysis is demonstrated by an unadjusted OR = 0.7347 (95% CI: [0.4371; 1.2348)], P = 0.2445, which did not differ much from the adjusted OR = 0.7328 (95% [0.4427; 1.2128] P = 0.2265. Moreover, the test for residual selection bias yielded a P-value = 0.4338.

## 4 Discussion

Our analyses found that the proportion of cardiotoxicity credited to nivolumab was the highest among ICI drugs; moreover, the probability of developing cardiotoxicity for someone treated with pembrolizumab was slightly lower than if the drug was nivolumab, but the difference was not statistically significant. However, although the results of the overall meta-analysis seemingly depicted a trend that favored pembrolizumab over nivolumab, it remained statistically insignificant even after performing subgroup analysis. The results remained inconclusive, and we observed that the considerable heterogeneity noted can have been because of the quality of studies, their designs, the great variation of outcome effect size, sample size, and other confounding factors such as cardiovascular history and type of cancers. With the exception of a subset of patients whose cardiotoxicity type was myocarditis, it can be observed that pembrolizumab therapy had a greater risk for cardiotoxicity than nivolumab.

Programmed cell death receptor inhibitor 1 is a protein found on T cells, which can bind to another ligand called PDL-1, thus preventing the T-cell-mediated destruction of other cells (18). Based on this mechanism, inhibitor agents for PD-1 and PDL-1 proteins have been manufactured to increase the T cells’ ability to fight cancer cells and increase overall survival (19, 20). Furthermore, there has been substantial objective improvement in cancer outcomes with the increased use of these novel agents, and it has now become a trend in the oncology field (21–23). ICI drugs from the PD1 receptor inhibitor subclass are well known to be associated with side effects such as pneumonitis, pruritus, and neurological, endocrine, and gastrointestinal adverse effects, although it has been observed that the frequency of the irAEs was relatively lower than with the other subclasses (23–25, 51). However, the PCD receptor inhibitor subclass has recently been identified in previous studies as bearing a certain cardiotoxicity adverse effect potential, albeit accounting for<5% of all adverse effects.

The cardiotoxicity spectrum has in majority been represented by myocarditis (26, 27), but other cardiac conditions can also be observed such as arrhythmias, pericardial effusion, heart failure, coronary events, pericarditis, and heart block (28). Existing case studies and pharmacovigilance data showed that the irAEs mainly affect cardiac conduction and myocyte function, which would then lead to heart damage (29, 30). It also appears that cardiotoxicity incidences are more observable among patients undergoing combination therapy (31). The routinely and broadly used PD1 ICI are pembrolizumab and nivolumab. Hence, the core objective was to identify whether there were any differences between them regarding cardiac adverse effects despite belonging to the same subclass. Can those differences also be related to their clinical efficacy, structure, mechanisms of action, spectrum, and frequency of side effects? Moreover, with regard to the cardiotoxic adverse effects, which one can be better than the other? The two drugs belong to the same subclass and are used for similar therapeutic indications, the most common being non-small cell lung cancers, melanoma, and metastasis (20). However, from a previous report, it seemed as though nivolumab was potentially a more cardiotoxic ICI, as roughly 60% of cardiotoxicity reports in cancer-treated patients were associated with nivolumab (32, 33) in mono or combination therapy regimen, but this was observed more in studies in which the quality on the NOS scale was not satisfactory; however, recent cohort and case–control studies (also included in this meta-analysis) rather reported an increased risk associated with the use of pembrolizumab (see Zachary et al., 2022). The results after pooling all the included studies showed that although there were more reports of cardiotoxicity for nivolumab, the difference in cardiotoxicity odds ratio between pembrolizumab and nivolumab was not statically significant. This was consistent with some previously reported results. One study that aimed to compare the efficacy of the two drugs (50) reported in their survival analysis that despite the observation of a higher objective response rate with pembrolizumab than nivolumab, there was no significant difference in the progression of free survival between the patients treated with pembrolizumab and those treated with nivolumab (22). Another investigation on the overall incidence and risk of irAE between the two drugs (95% CI: 0.97–1.79) indicated that the difference was not statistically significant (33, 34). Moreover, there were no significant discernible differences in the mechanism of action of the two drugs: pembrolizumab and nivolumab are both humanized IgG4 monoclonal antibodies against PD-1, but with the distinction that they do not induce antibody-dependent cellular cytotoxicity, as would be the case for normal IgG antibodies; hence, their toxicities and side effects have been more characterized as immune-related than cytotoxic (35). Analysis of the included studies showed that nivolumab had the highest proportion of induced cardiotoxicity among all ICI-induced cardiotoxicities (13). In addition, the majority of patients with reports of cardiotoxicity had other factors such as conventional cardiovascular risk factors or cardiovascular history. Although this may explain the frequency of reports of cardiotoxicity, it cannot explain the large disparity in the proportions of cardiotoxicity between the two drugs, given that the distributions of cardiovascular risk factors or the history of the two groups were comparable (36). In this study, cardiotoxicity refers to any cardiac damage irrespective of extent or severity. The cardiotoxicity odds ratio between pembrolizumab and nivolumab was not statistically significant; however, this does not imply that there would not be any difference at all. Therefore, after consideration of the descriptive proportions reported for both drugs, it can be suggested that nivolumab is associated with more cardiotoxicity events than pembrolizumab. One plausible explanation for this finding can be that nivolumab was introduced first and gained recognition first before pembrolizumab. Another possible theory would be that because in general, the overall number of cases with cancer expressing high PDL1 (expression: >50%) seems to be lower than that of cases with a low PDL1 expression (expression: 1–49%). Per standard recommendation, pembrolizumab is known to be effective in tumors expressing PDL1 at >50% and nivolumab in tumors expressing PDL1 at >1%. Therefore, there would naturally be more cases (low PDL1 expression) of cancer treated with nivolumab than with pembrolizumab. All these could have led to more reports of cardiac toxicity events with nivolumab than with pembrolizumab. Additionally, the small number of included studies and the high degree of heterogeneity between studies may have also played a role in the lack of statistical significance from the pooled odds ratio of cardiotoxicity effect between the two drugs. A few case series on ICI-induced cardiotoxicity also highlighted that cardiomyopathy, myocarditis, and conduction abnormalities were being underreported, which could have as well influenced the overall effect result of the current analysis (28). Another possibility can be that those considered to be developing cardiac toxicity were patients with cardiac clinical symptoms, resulting in non-involvement of the subclinical cases, which could have significantly influenced the results.

Numerous studies have shown that despite being relatively uncommon and few, the majority of myocarditis cases would present during the acute phase of therapy, with a propensity for seriousness and mortality, or the development of MACEs such as cardiac arrest, cardiac death, or stroke, but respond well to high corticosteroids for remission when administered in a timely manner (37–39). Although cardiac irAEs with ICIs are uncommon, the increased rate of mortality seen is an important factor to consider, as was also highlighted in another similar analysis (27). According to Dolladile et al.’s 2020 study, heart failure with left ventricular systolic dysfunction was seen among cases as a late adverse event. Therefore, patients treated with ICI should be monitored for at least 304 days. Additionally, because silent toxicities are possible (toxicity that manifests slowly before symptoms become obvious), such patients should also undergo routine cardiovascular screening for early detection of any abnormalities, especially for those aged >65 years and presenting at least two conventional cardiovascular risk factors or cardiovascular history (40). Nevertheless, the presumed advantages of early detection of cardiotoxicities through active screening, serial electrocardiograms, troponins, BNP, and echocardiography (which are helpful tools for the detection of subclinical cardiotoxicity during oncology therapy) should take into account the cost of testing as well as the possibility of false results, incorrect interpretation, and other related errors (31, 41). In a study, the highly sensitive troponin’s prognostic value showed that a value higher than 14 ng/L before the administration of pembrolizumab was significantly associated with a high incidence of MACE, including stroke and cardiac death (42). However, this does not necessarily mean that highly sensitive troponin should be considered an eligibility criterion for pembrolizumab or nivolumab therapy, but rather it can be useful as a predictor of cardiotoxicity risk.

This study has some limitations. The included studies were observational in nature, with some data collected from electronic and registered databases, implying a high susceptibility to information and selection biases (43). Selection bias was a concern in the (39) study, which was of a retrospective design, because there was no prospective cardiovascular screening protocol across all sites, and screening for cardiac biomarkers and other tests was left to the discretion of each individual care provider. (26) study was distinguished by the small sample size, which resulted in confounder interference and reporting bias. The criteria for control groups in two of the included studies were dubious, making it difficult to select a group of cancer patients with similar cardiovascular comorbidity and who underwent adequate testing to exclude cardiac pathology, as controls (28, 44). The criteria for selecting pembrolizumab or nivolumab for patients were not detailed or obvious in the results of the included studies. The risk of bias across the included studies could not have negated the evidence found in each study, but it could have led to an underestimation of association or effect size. According to two VigiBase analyses, pharmacovigilance analyses generate hypotheses that must be tested, ideally in prospective studies. Adverse Reaction Reporting System databases may be biased given the significant overlap of Individual Case Safety Reports (ICSRs) between databases. In addition, some cases may not have been reported to State drug enforcement authorities (34). The only included prospective cohort study had a short follow-up period, which may have led to a limited number of events and biased interpretation of results (25). In addition, randomized clinical trials on cardiotoxicity issues were not available at the time of the search, resulting in a limited number of studies, small sample sizes, and low statistical significance. Moreover, unpublished records on the topic as well as articles published in languages other than English cannot be evaluated. To our knowledge, this is the first meta-analysis to directly compare the two drugs, and hence, these results are still important, because they highlight the need for additional, in-depth research on this topic in multiethnic, large-center settings to provide oncology patients with the best possible care while reducing the likelihood of cardiotoxicity.

## 5 Conclusion

Immune checkpoint inhibitors have revolutionized the treatment of advanced-stage cancers including metastases; however, the potential danger to vital organs (52) such as the heart cannot be overlooked. Therefore, it is paramount to look at every strategy with the potential to limit, reduce, or control the magnitude of this issue. Previous studies regarding the cardiotoxicity risks of ICIs and comparisons between anti-PD1 and anti CTLA4 (45) were made; however, this paper addressed the direct comparison between nivolumab and pembrolizumab cardiotoxicity potentials. Contrary to what was recently reported (46, 47), the descriptive proportions described herein have provided a clear indication that nivolumab-induced cardiotoxicities are reported more in the literature over the past years than pembrolizumab-induced cardiotoxicities. The discrepancy between these findings and previous ones highlights the need for a prospective analysis on a larger sample cohort. However, the consensus on the need for proper cardiac screening before and after remains strong among researchers (48, 49). Therefore, the importance of multidisciplinary collaboration between oncologists, immunologists, and cardiologists in the management of cancer patients cannot be overemphasized.

## Data availability statement

The original contributions presented in the study are included in the article/**Supplementary Material**. Further inquiries can be directed to the corresponding author.

## Author contributions

This manuscript was compiled and written by FN under the direction, guidance, and supervision of Z-QW. Proofreading and editing were done by CM. All authors contributed to the article and approved the submitted version.
