# Supplementary material for: Comparative cardiotoxicity risk of pembrolizumab versus nivolumab in cancer patients undergoing immune checkpoint inhibitor therapy: A meta-analysis
Source: Front Oncol. 2023 Mar 29;13:1080998. doi: 10.3389/fonc.2023.1080998 (PMC10090546; doi:10.3389/fonc.2023.1080998)
Supplement: Supplementary file 1 [file DataSheet_1.docx]

1.
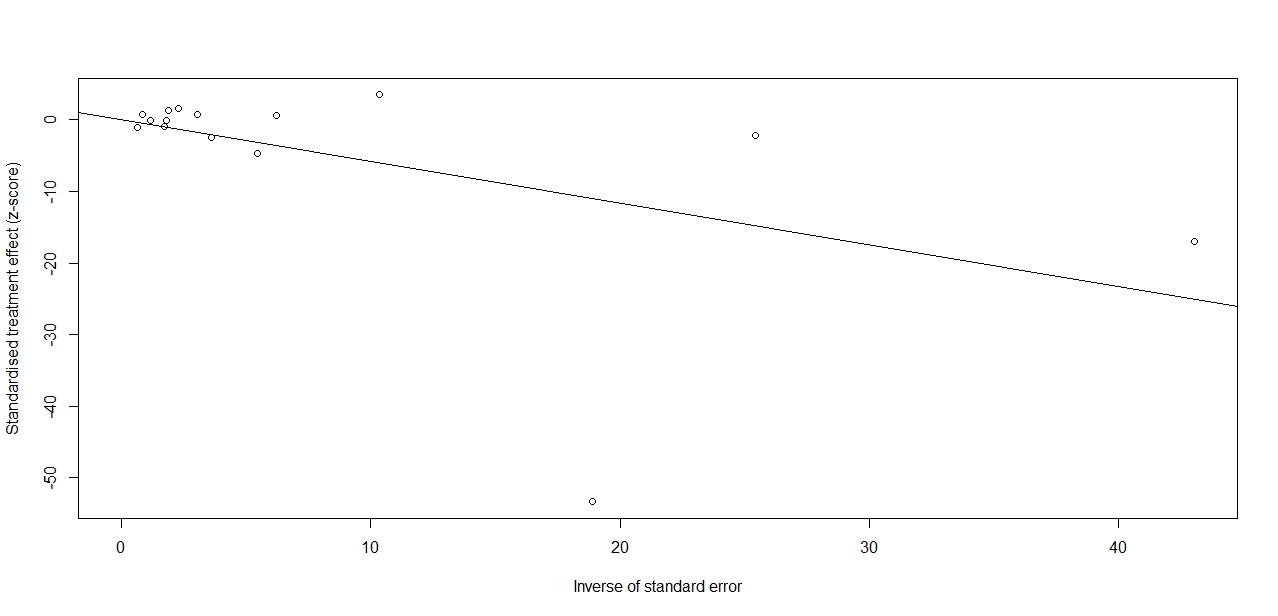

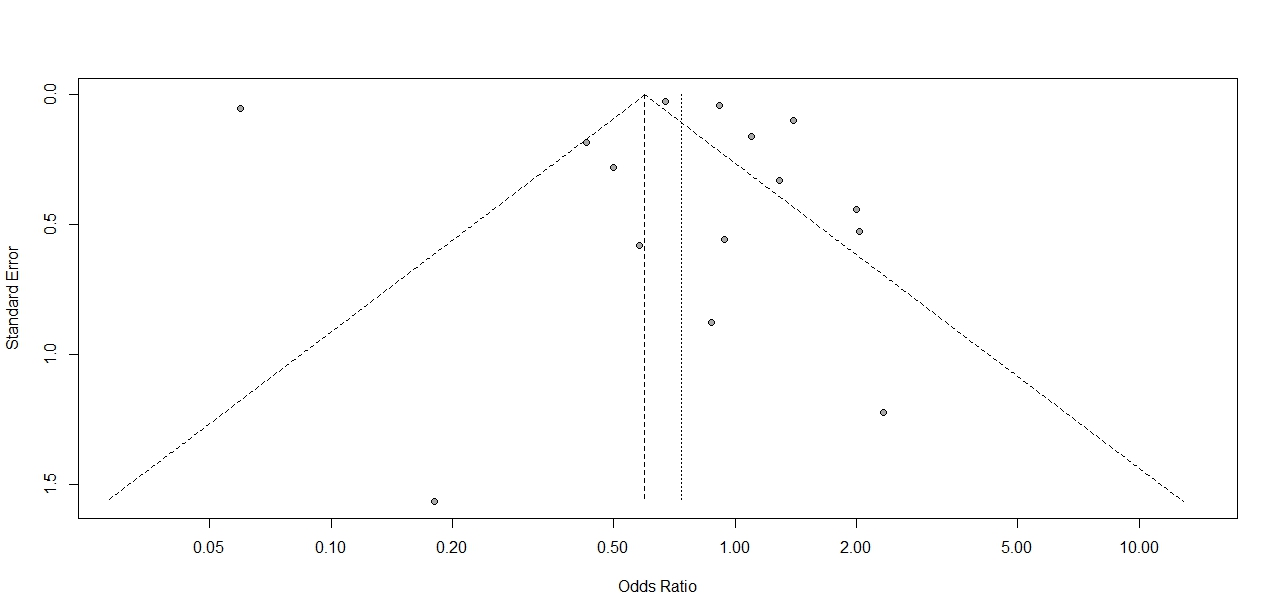
Overall analysis of Funnel plot

Linear regression test of funnel plot asymmetry

Test result: t = -0.00, df = 13, p-value = 0.9963

Sample estimates:

bias se.bias intercept se.intercept

-0.0194 4.1039 -0.5808 0.2868

1. Sensitivity analysis under copas selection model analysis

p.publ OR 95%-CI p.trt p.rsb N

1.0000 0.7328 [0.4427; 1.2128] 0.2265 0.4338 0

0.9569 0.7179 [0.4335; 1.1890] 0.1979 0.4997 0

0.9405 0.7118 [0.4301; 1.1780] 0.1860 0.5372 1

0.9054 0.6977 [0.4238; 1.1486] 0.1570 0.6332 1

0.8732 0.6839 [0.4163; 1.1233] 0.1334 0.7476 2

0.8435 0.6704 [0.4096; 1.0974] 0.1118 0.9358 2

0.8151 0.6571 [0.4031; 1.0714] 0.0923 1.0000 3

0.7878 0.6439 [0.3968; 1.0450] 0.0748 1.0000 3

0.7625 0.6313 [0.3913; 1.0185] 0.0594 1.0000 4

0.7386 0.6190 [0.3872; 0.9897] 0.0452 1.0000 4

0.7145 0.6064 [0.3867; 0.9507] 0.0292 1.0000 5

0.6929 0.5948 [0.4052; 0.8730] 0.0080 1.0000 5

0.6889 0.5925 [0.4199; 0.8361] 0.0029 1.0000 5

Adjusted estimate 0.7328 [0.4427; 1.2128] 0.2265 0.4338 0

Unadjusted estimate 0.7347 [0.4371; 1.2348] 0.2445

Significance level for test of residual selection bias: 0.1

min max

range of gamma0: -0.2694 2.0000

range of gamma1: 0.0000 0.0304

Largest standard error (SE): 1.5664

Range of probability publishing trial with largest SE:

min max

0.3938 0.9783

Calculation of orthogonal line:

level nobs adj.r.square slope se.slope

-0.54 27 -0.03037873 1.206108 2.49631003

-0.52 23 0.82306059 5.904204 0.58081092

-0.52 20 0.99972604 -30.717559 0.11665617

-0.50 21 0.99828108 -29.230201 0.27120618

-0.48 21 0.99945060 -30.660061 0.16073732

-0.46 20 0.99972212 -28.975891 0.11082734

-0.44 21 0.99718259 -28.502046 0.33874097

-0.42 21 0.99656780 -29.183754 0.38293178

-0.40 21 0.99722851 -28.999699 0.34182803

-0.38 21 0.99526972 -27.609376 0.42556241

-0.36 20 0.99973956 -26.351382 0.09757472

-0.34 20 0.99976850 -25.365073 0.08854807

Legend:

p.publ - Probability of publishing study with largest SE

p.trt - P-value for test of overall treatment effect

p.rsb - P-value for test of residual selection bias

N - Estimated number of unpublished studie


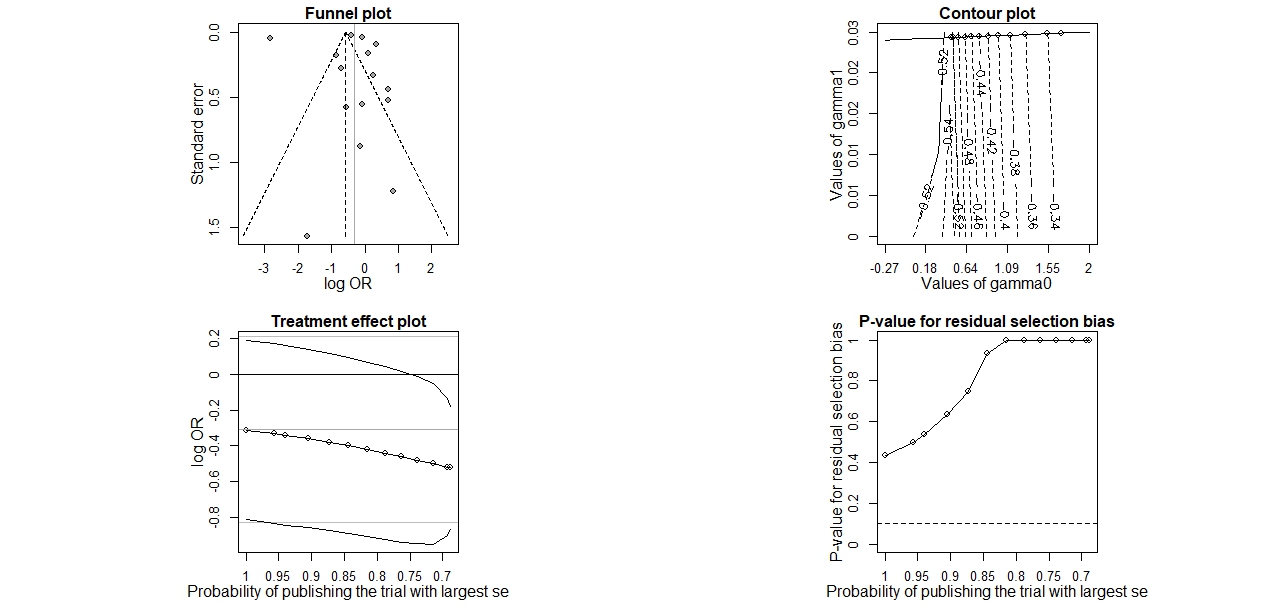


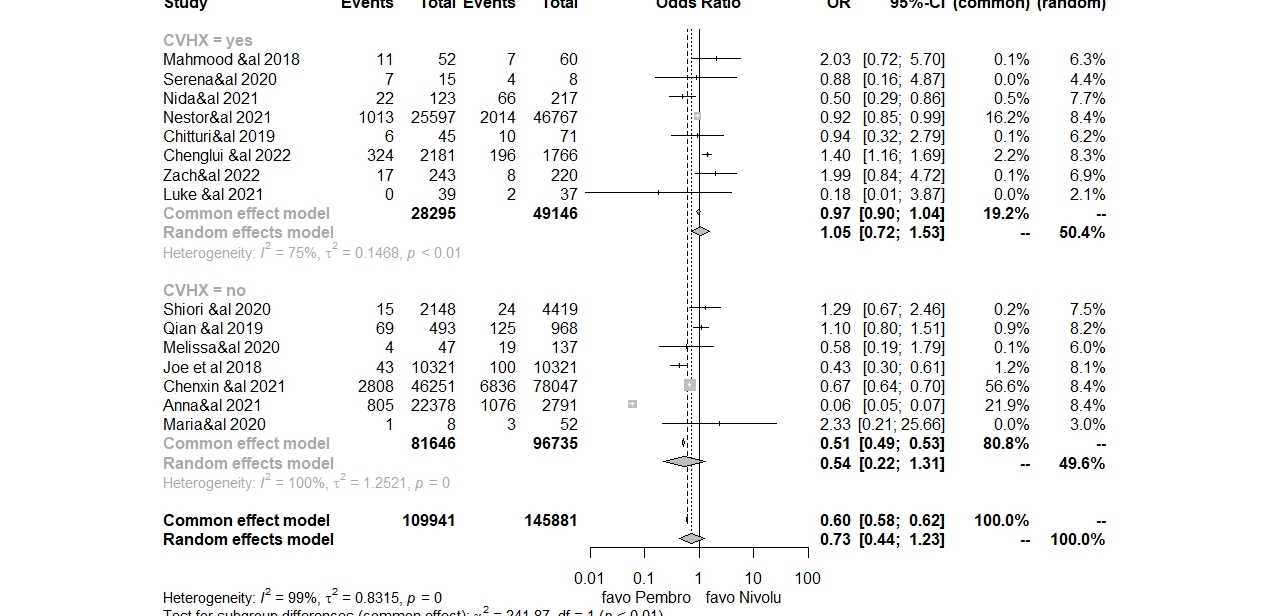

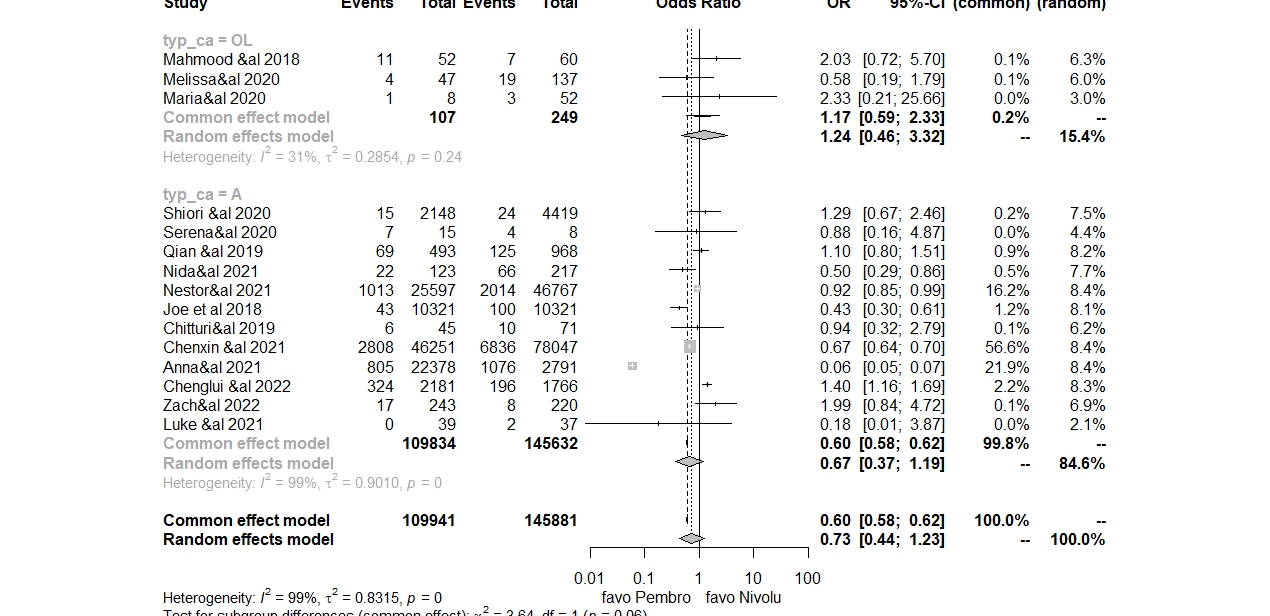
3-subgroup analyses forest plots


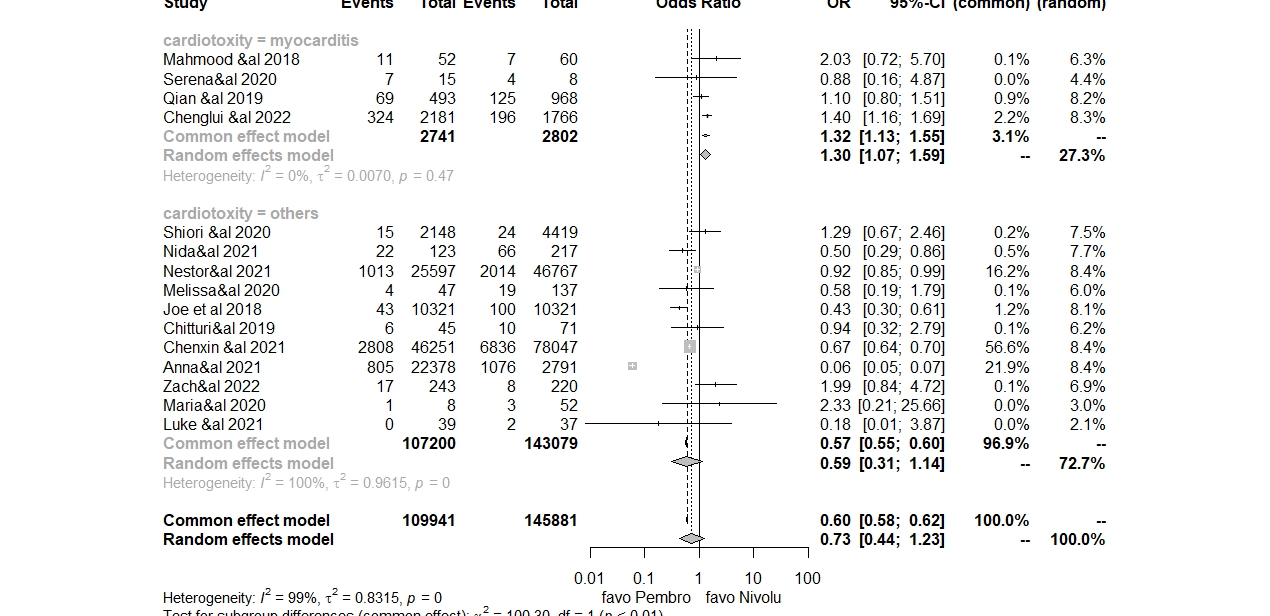

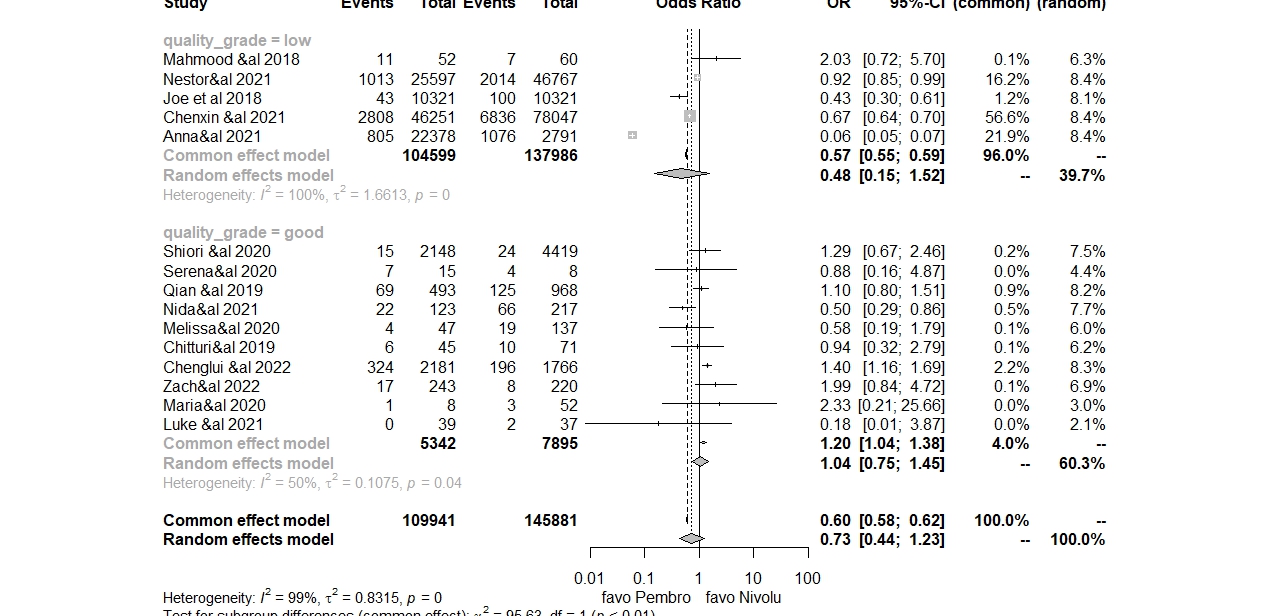


Table showing the distribution of Myocarditis, Pericarditis and arrhythmia across included studies

| Study | year | Myocarditis | | | Pericarditis | | | Arrhythmia | | |
| --- | --- | --- | --- | --- | --- | --- | --- | --- | --- | --- |
|  |  | T.e & prop | P.e & prop | N.e & prop | T.e, & prop | P.e & prop | N.e & prop | T.e & prop | P.e & prop | N.e & prop |
| Mahmood &al | 2018 | 35 | 11 (31.0) | 7 (20.0) | NR | NR |  |  |  |  |
| Shiori &al | 2020 | 229 | 15 | 24 | NR | NR |  |  |  |  |
| Serena&al | 2020 | 13 | 8 | 4 | NR | NR |  |  |  |  |
| Qian &al | 2019 | 315 | 69 (21.90) | 125 (39.68) |  |  |  |  |  |  |
| Nida&al | 2021 | 1 (0.24) | 0 | 1 (0.46) | 8 (1.89) | 0 | 7 (3.23) | 26 (6.13) | 0 | 15 (6.91) |
| Nestor&al | 2021 | 839 | 197 | 399 | 335 | 97 | 147 | 561 | 107 | 234 |
| Melissa&al | 2020 | 9 (39%) |  |  | 4 (17%) |  |  | 7 (31%) |  |  |
| Joe et al | 2018 | 122(0.39%) | 22 (18.0) | 58 (47.5) | 95(0.3%) | 21 (22.1) | 42 (44.2) | 37 (0.12%) |  |  |
| Chitturi&al | 2019 | 44% |  |  | 33.3% |  |  | 48% |  |  |
| Chenxin &al | 2021 | 614 |  | 24 | 423 |  |  | 576 |  |  |
| Anna&al | 2021 | 446 | 171 (16.3%) | 215 (16.4%) | 204 | 87 (8.3%) | 81 (6.2%) | 194 | 71 (6.8%) | 95 (7.3%) |
| Chenglui &al | 2022 | 691) | 324  46.9% | 196 28.4% |  |  |  |  |  |  |
| Zach&al | 2022 | 8(1.5%) | 0 | 0 | 12(2.2%) | 7(2.9%) | 5(2.3%) | 14(2.6%) | 8(3.3%) | 5(2.3%) |
| Maria&al | 2020 |  |  |  | 4 | 1 | 3 |  |  |  |
| Luke &al | 2021 |  |  |  |  |  |  | 4 | 0 | 2 |

SEARCH STRATEGY

The entry terms were: Keytruda or Pembrolizumab; PD1 inhibitors; anti-PD1 drugs; Nivolumab or Opdivo; cardiotoxicities or cardiac toxicity; toxicities.

Our initial search strategy on Pubmed was (((Keytruda) OR (pembrolizumab)) AND ((cardiotoxicities) OR (cardiac toxicity))) AND (((Opdivo) AND (Nivolumab)) AND ((cardiotoxicities) OR (cardiac toxicity))), which yielded few results. Therefore, another search strategy was implemented as follows (immune checkpoint inhibitors) AND (cardiotoxicity) which brought more results and was replicated in Embase and google scholar too.
